# Intradermal penicillin skin test is more likely to be positive compared to prick skin test in patients with a history of penicillin allergy

**DOI:** 10.1186/2045-7022-4-S3-P101

**Published:** 2014-07-18

**Authors:** Miguel Park

**Affiliations:** 1 Mayo Clinic, USA

## Background

Penicillin skin testing (PST) with the major determinant (benzyl-penicilloyl) and minor determinant mix (penicillin G, benzyl-penicilloate, and benzyl-penilloate) is one of the major diagnostic tools in the evaluation of a penicillin allergy mediated by IgE. The PST begins with a skin prick test (SP) with the above components and, if negative, then proceeds to an intradermal testing (ID). However, it is unclear in the literature, how often the PST is positive to SP compared to ID testing. Hence, we conducted this study to determine the rates of positive PST to SP compared to ID skin test.

## Methods

Basic demographics (gender and age) and rates of positive ID PST compared to SP PST were determined in patients with a history of penicillin allergy undergoing penicillin allergy evaluation from June 1, 2002 to June 30, 2004. A wheal and flare 3x3mm greater than the negative control was defined as a positive PST. The gender and rates of ID versus SP PST were presented as percentages and the age in years. Using Fisher's exact test, we compared the difference in the proportion of males and females who had a positive SP vs. ID PST. P 0.05 was considered statistically significant. The IRB approved the study and all subjects signed a written informed consent.

## Results

1,759 patients with a history of penicillin allergy underwent PST. The mean age (SD) was 60 ¡À 15 years (5517 years in the positive PST group versus 60 15 years in the negative PST group). Among the positive PST, 53 (83%) were females and 11 (17%) were males. 64 of 1759 (4%) had a positive PST to either SP or ID testing. Among the positive PST patients, 3 of 64 (5%) had a positive SP compared to 61 (95%) ID skin testing. Gender did not appear to increase the rates of positive ID PST compared to SP [2 of 3 (66%) females were positive to SP PST compared 51 of 61 (84%) females were positive ID PST; p=0.44].

## Conclusion

ID PST is more likely to be positive compared to SP PST in patients with a history penicillin allergy.

